# The Association Between Perivascular Spaces and Cerebral Blood Flow, Brain Volume, and Cardiovascular Risk

**DOI:** 10.3389/fnagi.2021.599724

**Published:** 2021-08-31

**Authors:** Sirui Liu, Bo Hou, Hui You, Yiwei Zhang, Yicheng Zhu, Chao Ma, Zhentao Zuo, Feng Feng

**Affiliations:** ^1^Department of Radiology, Peking Union Medical College Hospital, Chinese Academy of Medical Sciences and Peking Union Medical College, Beijing, China; ^2^Department of Neurology, Peking Union Medical College Hospital, Chinese Academy of Medical Sciences and Peking Union Medical College, Beijing, China; ^3^Department of Human Anatomy, Histology and Embryology, School of Basic Medicine, Peking Union Medical College, Beijing, China; ^4^State Key Laboratory of Brain and Cognitive Science, Institute of Biophysics, Chinese Academy of Sciences, Beijing, China; ^5^Sino-Danish College, University of Chinese Academy of Sciences, Chinese Academy of Sciences, Beijing, China

**Keywords:** enlarged perivascular spaces, basal ganglia, magnetic resonance imaging, cerebral blood flow, gray matter volume, cardiovascular risk burden

## Abstract

**Background:** Basal ganglia perivascular spaces are associated with cognitive decline and cardiovascular risk factors. There is a lack of studies on the cardiovascular risk burden of basal ganglia perivascular spaces (BG-PVS) and their relationship with gray matter volume (GMV) and GM cerebral blood flow (CBF) in the aging brain. Here, we investigated these two issues in a large sample of cognitively intact older adults.

**Methods:** A total of 734 volunteers were recruited. MRI was performed with 3.0 T using a pseudo-continuous arterial spin labeling (pCASL) sequence and a sagittal isotropic T1-weighted sequence for CBF and GMV analysis. The images obtained from 406 participants were analyzed to investigate the relationship between the severity of BG-PVS and GMV/CBF. False discovery rate-corrected *P*-values (*P*_FDR_) of <0.05 were considered significant. The images obtained from 254 participants were used to study the relationship between the severity of BG-PVS and cardiovascular risk burden. BG-PVS were rated using a 5-grade score. The severity of BG-PVS was classified as mild (grade <3) and severe (grade ≥3). Cardiovascular risk burden was assessed with the Framingham General Cardiovascular Risk Score (FGCRS).

**Results:** Severe basal ganglia perivascular spaces were associated with significantly smaller GMV and CBF in multiple cortical regions (*P*_FDR_ <0.05), and were associated with significantly larger volume in the bilateral caudate nucleus, pallidum, and putamen (*P*_FDR_ <0.05). The participants with severe BG-PVS were more likely to have a higher cardiovascular risk burden than the participants with mild BG-PVS (60.71% vs. 42.93%; *P* =0.02).

**Conclusion:** In cognitively intact older adults, severe BG-PVS are associated with smaller cortical GMV and CBF, larger subcortical GMV, and higher cardiovascular risk burden.

## Introduction

Perivascular spaces, also known as Virchow–Robin spaces, are interstitial fluid-filled spaces surrounding the wall of small penetrating vessels (Arbel-Ornath et al., 2013; Yakushiji et al., 2014). Accumulating evidence has shown that the severity of basal ganglia perivascular spaces (PVS) (BG-PVS) is associated with vascular abnormalities and vascular cognitive decline (Kalaria, 2012; Banerjee et al., 2017; Duperron et al., 2018). BG-PVS can also be observed in elderly individuals (Zhu et al., 2010). However, the significance in an aging brain remains poorly understood. In addition, the relationship between the severity of BG-PVS and regional gray matter volume (GMV) and gray matter cerebral blood flow (CBF) remains unclear. A community-based study (Zhu et al., 2010) revealed that PVS are not associated with visually inspected brain atrophy and does not yield brain volume quantification. Therefore, the relationship between BG-PVS and the quantified volume of different brain regions remains to be explored further. CBF, which reflect hemodynamic alterations, is an imaging biomarker for the identification of vulnerable brain regions. Since BG-PVS are considered to be caused by vascular abnormalities (Kress et al., 2014; Kyrtsos and Baras, 2015; Ramirez et al., 2016), it was hypothesized that individuals with BG-PVS could have alterations in CBF and GMV. Previous studies have shown that BG-PVS are associated with various cardiovascular risk factors (such as age, sex, hypertension, and arteriolosclerosis) (Zhu et al., 2010; Aribisala et al., 2014). It is well established that cardiovascular risk factors are interrelated, making it difficult to isolate their individual effects on BG-PVS. The Framingham General Cardiovascular Risk Score (FGCRS), which combines multiple cardiovascular risk factors with demographic data, can be used to assess cardiovascular risk burden (D'Agostino et al., 2008). However, to the best knowledge of the authors, studies that link BG-PVS with cardiovascular risk burden are lacking. Hence, this study extended the research mentioned above and carried out cross-sectional estimation of cognitively intact older adults to determine whether GMV and CBF are associated with the severity of BG-PVS. This study also aimed to evaluate the relationship between BG-PVS and cardiovascular risk burden.

## Materials and Methods

### Participants

This study was approved by the institutional review board of Peking Union Medical College Hospital (PUMCH), and a written informed consent was obtained from all the participants, who were recruited from two ongoing cohort studies (cohort A and cohort B). Cohort A is a community-based study that comprises elderly subjects from Beijing, China. Cohort B is a large-scale willed body donation program in the Chinese Academy of Medical Sciences and PUMCH (Zhang et al., 2018).

**Figure 1:**
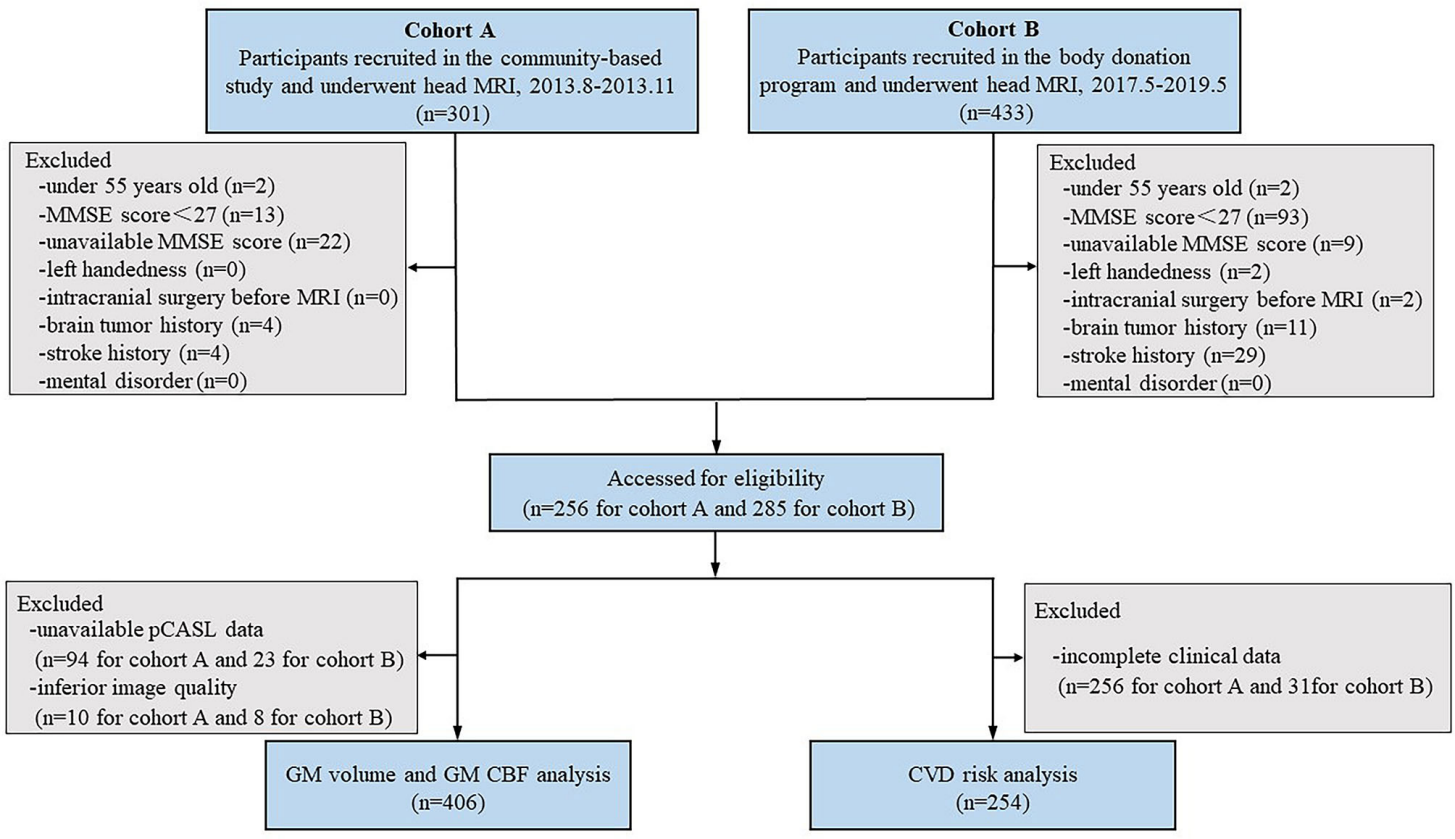
Study flowchart shows the recruitment of study participants. MMSE, Mini-Mental State Exam; pCASL, pseudo-continuous arterial spin labeling.

Figure 1 shows the flowchart of participant enrollment. This cross-sectional study included 734 participants (*n* = 301 for cohort A and *n* = 433 for cohort B). The exclusion criteria of participants were as follows: (1) under 55 years old; (2) a Mini-Mental State Exam (MMSE) score of <27 who might have apparent cortical hypoperfusion and atrophy (Schuff et al., 2009; Gao et al., 2013; Sun et al., 2016; Ten Kate et al., 2018); (3) an unavailable MMSE score; (4) left-handed participants; (5) a history of intracranial surgery before MRI; and (6) with brain tumor, stroke, and mental disorder. Thus, a total of 193 participants were excluded. The remaining 541 participants (*n* = 256 for cohort A and *n* = 285 for cohort B) were eligible for the subsequent analysis. Participants with inferior image quality (*n* = 18), unavailable pseudocontinuous arterial spin labeling (pcASL) data (*n* = 117), and/or incomplete clinical data (*n* = 287) were also excluded. Finally, the MRI images obtained from 406 participants were used to investigate the relationship between the severity of BG-PVS and GMV/CBF, while the MRI images obtained from 254 participants were used to investigate the relationship between the severity of BG-PVS and cardiovascular risk burden.

### MRI Acquisition

The magnetic resonance imaging studies of cohort A were performed with a 3.0 T GE MRI scanner (GE Discovery MR750, GE Healtchare, Milwaukee, WI, United States). The MRI protocols used with cohort A comprised three serial sequences: an axial T2-PROPELLER sequence (TR/TE = 7,912/92 ms; slice thickness = 4 mm; and FOV = 256 × 256 mm^2^), an axial pCASL sequence (TR/TE = 4,886/10.5 ms; FOV = 240 × 240 mm^2^; slice thickness = 4 mm; post-labeling delay = 2,025 ms; labeling duration = 1,450 ms), and a sagittal 3D BRAVO sequence (TI/TR/TE = 400/6.9/2.6 ms; FOV = 256 mm^2^ × 256 mm^2^; slice thickness = 1. mm; matrix size = 256 × 256). The MRI studies of cohort B were performed with another 3.0 Tesla GE MRI scanner (GE SIGNA PET/MR, GE Healthcare Systems, Chicago IL, United States). The MRI protocols used for cohort B were as follows: an axial T2-PROPELLER sequence (TR/TE = 3,324/81 ms; slice thickness = 4 mm; and FOV = 256 × 256 mm^2^), an axial pCASL sequence (TR/TE = 4,874/10.7 ms; FOV = 240 × 240 mm^2^; slice thickness = 4 mm; post-labeling delay = 2,025 ms; labeling duration = 1,450 ms), and a sagittal T1-weighted 3D BRAVO sequence (TI/TR/TE = 450/7.4/3.2 ms; FOV = 256 mm^2^ × 256 mm^2^; slice thickness = 1.1 mm; matrix size = 256 × 256). The MR sequences and parameters used with cohort B were similar to those used with cohort A, which could greatly reduce the potential differences between the MRI scanners (Lundervold et al., 2000; Mutsaerts et al., 2015; Liu et al., 2020).

### ASL Data Process

CBF maps of ASL were generated on GE AW 4.5 workstation by a software 3D ASL Functool kit (Lin et al., 2018). This software processes pcASL data in a standardized one-click mode. The same model used for CBF calculation was based on a previous study (Williams et al., 1992; Lin et al., 2020). Data were preprocessed using SPM12 (Wellcome Department of Cognitive Neurology, Institute of Neurology, London, United Kingdom) implemented on MATLAB (MathWorks, Natick, MA, United States). First, the CBF maps were co-registered to 3D T1-weighted images. Second, the 3D T1-weighted images were segmented into the gray matter, white matter, and cerebrospinal fluid (CSF) based on tissue probability maps in each voxel and then normalized into a Montreal Neurological Institute (MNI) template. After that, the spatially normalized images were smoothed with an isotropic Gaussian kernel filter of 6 mm full width at half maximum (FWHM). The MNI-normalized smoothed gray matter was used to create a gray matter mask with a threshold value of 0.5. Finally, the Hammers atlas was used to extract the CBF of 68 brain regions.

### Anatomic Morphometric Analysis

A morphometric analysis based on the 3D T1-weighted images was performed using CAT12 implemented on SPM12 (Wellcome Department of Cognitive Neurology, Institute of Neurology, London, United Kingdom) (Farokhian et al., 2017). The standard pipeline was first used to segment the 3D T1-weighted images into gray matter, white matter, and CSF. After that, the 3D T1-weighted images were normalized to the MNI template and smoothed with an isotropic Gaussian kernel filter of 8 mm FWHM. Then, the Hammers atlas was used to extract the GMV of 68 brain regions (Rodionov et al., 2009; Yaakub et al., 2020).

### Rating of MRI-Visible BG-PVS

**Figure 2:**
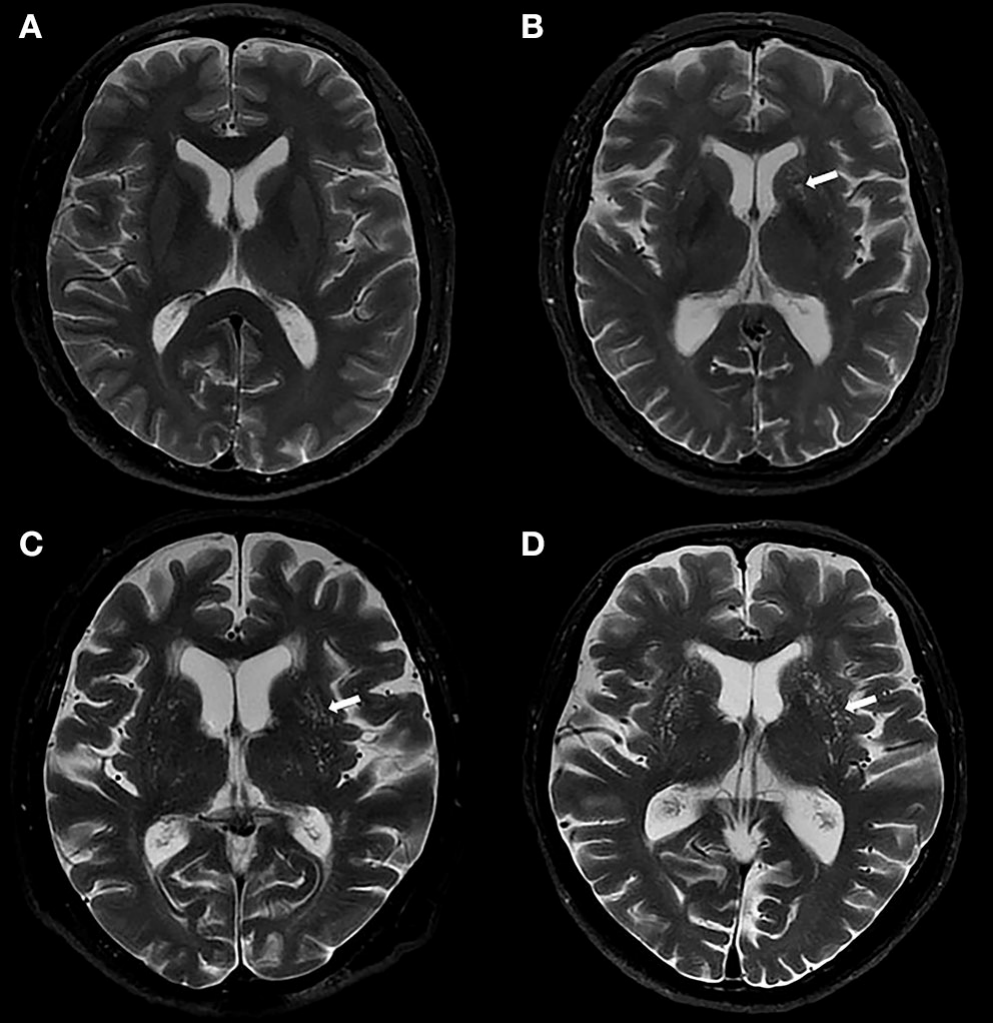
Examples of MRI-visible BG-PVS. **(A,B)** Axial T2-weighted images show the mild grade of BG-PVS (arrow), **(C,D)** Axial T2-weighted images show the severe grade of BG-PVS (arrow).

Basal ganglia perivascular spaces were identified according to the standards for reporting vascular changes on neuroimaging (Wardlaw et al., 2013). These were visually rated on axial T2-weighted images on a predefined slice (the first slice above the anterior commissure in the BG) (Banerjee et al., 2017) by a trained rater. Then, 82 (20%) individuals were randomly selected and visually assessed by another rater. Both raters were blinded to the clinical information. Only lesions that met the following conditions were regarded as BG-PVS: CSF-like signal lesions (hyperintense on T2 and hypointense on T1, and fluid-attenuated inversion recovery); lesions with a linear, round, or ovoid shape; lesions with a clear boundary; the maximum diameter of lesions <3 mm; lesions with no mass effect; and lesions in the areas supplied by perforating arteries (Zhu et al., 2010). Basal ganglia perivascular spaces were rated using a 5-grade score: grade 0 for no BG-PVS, grade 1 for <10 BG-PVS, grade 2 for 10 to 20 BG-PVS, grade 3 for 20 to 40 BG-PVS, and grade 4 for >40 BG-PVS (Doubal et al., 2010; Banerjee et al., 2017). Both hemispheres were counted, and the hemisphere with higher score was recorded. The number of individuals at each grade of BG-PVS varies greatly (Zhu et al., 2010; Yakushiji et al., 2014). Therefore, for statistical analysis, we categorized the severity of BG-PVS as mild (grade <3) or severe (grade ≥3). Figure 2 presents the BG-PVS examples for each severity grade.

### Assessment of Cardiovascular Risk Burden

We calculated FGCRS based on age, sex, high-density lipoprotein cholesterol, total cholesterol, systolic blood pressure (SBP), diabetes, and smoking history (D'Agostino et al., 2008). FGCRS is calculated by summing up the points from all of these risk factors. According to FGCRS, the cardiovascular risk burden was divided into two categories for statistical analysis, lower (FGCRS <17) and higher (FGCRS ≥17) (Song et al., 2020).

### Statistical Analysis

Statistical analysis was performed using SPSS (version 20, IBM Inc., Armonk, NY, United States). Categorical variables were tested by χ^2^ test, and were described in both percentage and frequency. Continuous variables were tested by Student's *t*-test or Mann–Whitney U test, and were described with mean and SD estimates. The inter-rater consistency of the severity of BG-PVS was evaluated with kappa (κ) value.

Using age, sex, scanner, and total intracranial volume (TIV) as covariates, the difference in GMV between the mild and severe BG-PVS groups was analyzed using univariate linear models. Using age, sex, scanner, and regional GMV as covariates, the difference in CBF between the mild and severe BG-PVS groups was analyzed using univariate linear models. The Benjamini–Hochberg procedure was performed to control for false discovery rate (FDR). FDR-corrected *P*-values (*P*_FDR_) of < 0.05 were considered significant. Logistic regression analysis was performed to assess the difference in cardiovascular risk burden between the mild and severe BG-PVS groups.

## Results

### Inter-rater Consistency

The inter-rater consistency was high for assessing the severity of BG-PVS (κ =0.883). The two raters reached a consensus on the classification of 81 images.

### Basal Ganglia Perivascular Spaces, Gray Matter Volume, and Cerebral Blood Flow

A total of 406 participants (169 men and 237 women, mean age: 69.44 ± 7.89 years old) were included in the GMV and CBF analysis (see Table 1 for the summary of details).

**Table 1:** Demographic characteristics of the overall MRI sample classified according to the severity of BG-PVS.

	**Mild BG-PVS group**	**Severe BG-PVS group**	**Statistical test**	***P* value**
Number	316 (77.83%)	90 (22.17%)		
Mean age at MRI (SD)	68.27 (7.45)	73.57 (8.03)	Student's t	<0.001^***^
Male (%)	120 (37.97)	49 (54.44)	χ2	0.005^**^
MMSE score	28.84 (1.06)	28.77 (1.02)	Mann-Whitney U	0.466

*BG-PVS, basal ganglia perivascular spaces; MMSE, Mini-Mental State Exam; SD, standard deviation.*

* *P < 0.05;*

** *P < 0.01;*

*** *P < 0.001*.

Among the 68 brain regions exacted from Hammers atlas, a total of 10 brain structures (e.g., ventricles and corpus callosum) that did not belong to the gray matter were excluded, and the remaining 58 brain regions were used for morphological and metabolic analysis.

**Figure 3:**
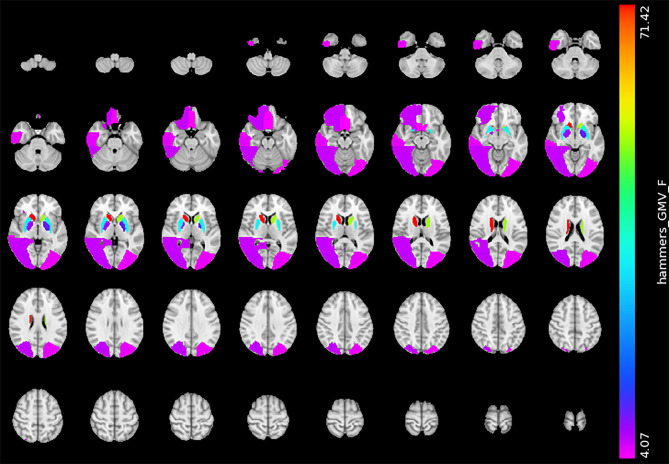
Significant difference in GMV between the mild BG-PVS group and the severe BG-PVS group. The severe BG-PVS group shows a significantly smaller volume in multiple cortical areas and a larger volume in the bilateral caudate nucleus, pallidum, and putamen (*P*_FDR_ < 0.05, corrected for age, sex, scanner and TIV). The F-score bar is shown on the right. The left part of the figure represents the individual's right side. GMV, gray matter volume; BG-PVS, basal ganglia perivascular spaces; TIV, total intracranial volume.

The gray matter volume analysis revealed that the severe BG-PVS group had a significantly smaller GMV than the mild BG-PVS group, in the bilateral lateral occipital lobe, bilateral rectus gyrus, right orbitofrontal gyrus, right posterior temporal lobe, and right inferior middle temporal lobe (*P*_FDR_ < 0.05, corrected for age, sex, scanner, and TIV, Figure 3) (see details in Supplementary Material 1). In addition, the severe BG-PVS group also showed a significantly larger volume in the bilateral caudate nucleus, pallidum, and putamen (*P*_FDR_ < 0.05, corrected for age, sex, scanner, and TIV, Figure 3).

**Figure 4:**
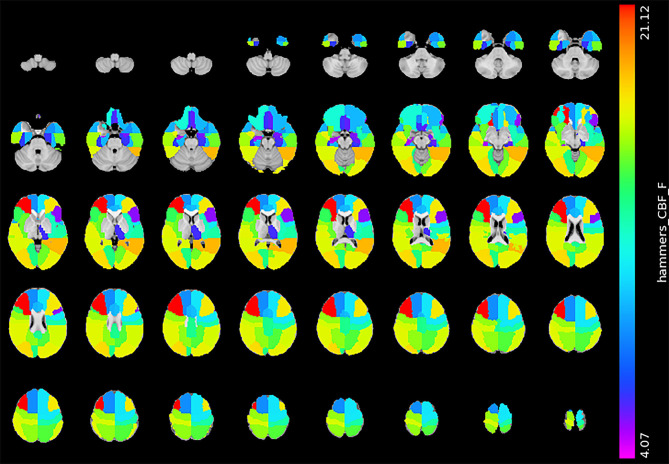
Significant difference in CBF between the mild BG-PVS group and the severe BG-PVS group. The severe BG-PVS group shows a significantly lower CBF in the cortex and left thalamus (*P*_FDR_ < 0.05, corrected for age, sex, scanner and regional GMV). The F-score bar is shown on the right. The left part of the figure represents the right side of the individual. CBF, gray matter cerebral blood flow; BG-PVS, basal ganglia perivascular spaces; GMV, gray matter volume.

Figure 4 shows the statistical result of the CBF. Compared with the mild BG-PVS group, the severe BG-PVS group showed widespread hypoperfusion in the cortex and left thalamus (*P*_FDR_ < 0.05, corrected for age, sex, scanner, and regional GMV, Figure 4) (see details in Supplementary Material 1).

### BG-PVS and Cardiovascular Risk Burden

A total of 254 participants (107 men and 147 women, mean age: 71.27 ± 7.77 years old) were included in the analysis between the severity of BG-PVS and cardiovascular risk burden. Table 2 presents the comparison of cardiovascular risk factors of all individuals stratified by the severity of BG-PVS. Severe BG-PVS were associated with male sex (*P* = 0.023) and age (*P* < 0.001). Those in the severe BG-PVS group were more likely to have hypertension (*P* = 0.032), higher SBP (*P* = 0.001), and higher total cholesterol (*P* = 0.001). The participants with severe BG-PVS were twice as likely to have a high cardiovascular risk burden than the participants with mild BG-PVS (*P* = 0.02; OR, 2.06; 95% CI, 1.12–3.77).

**Table 2:** Comparison of cardiovascular risk factors in subjects with mild or severe BG-PVS.

	**Mild BG-PVS group**	**Severe BG-PVS group**	**Statistical test**	***P* value**
Number	198 (77.95%)	56 (22.05%)		
Mean age (SD)	69.55 (7.07)	77.32 (7.14)	Student's *t*	<0.001^***^
Male (%)	76 (38.38)	31 (55.36)	χ^2^	0.023^*^
MMSE score	28.76 (1.07)	28.59 (0.93)	Mann-Whitney U	0.197
Higher cardiovascular risk burden (%)	85 (42.93)	34 (60.71)	Logistic regression	0.020^*^
Mean SBP mm Hg (SD)	132.53 (16.01)	140.91 (16.85)	Student's *t*	0.001^**^
TChol mg/dL (SD)	199.57 (39.79)	179.29 (43.98)	Student's *t*	0.001^**^
HDL-C mg/dL (SD)	51.87 (12.51)	55.05 (16.85)	Student's *t*	0.193
Smoker (%)	45 (22.73)	12 (21.43)	χ^2^	0.837
Hypertension (%)	106 (53.53)	39 (69.64)	χ^2^	0.032*
Diabetes (%)	41 (20.71)	11 (19.64)	χ^2^	0.862

*BG-PVS, basal ganglia perivascular spaces; MMSE, Mini-Mental State Exam; SD, standard deviation; SBP, systolic blood pressure; TChol, total cholesterol, HDL-C, high-density lipoprotein cholesterol.*

* *P < 0.05;*

** *P < 0.01;*

*** *P < 0.001*.

## Discussion

This study showed that BG-PVS were associated with cardiovascular risk burden and regional differences in CBF and GMV. This study advanced those of several groups (Kalaria, 2012; Banerjee et al., 2017; Duperron et al., 2018), which associated BG-PVS with vascular cognitive impairment with a larger population of cognitively intact individuals.

Previous MRI studies have shown no association between BG-PVS and whole-brain atrophy (Zhu et al., 2010, 2011; Yakushiji et al., 2014). However, a strong association between PVS and brain weight has been observed in a postmortem study (van Swieten et al., 1991). This study found that the severe BG-PVS group showed a significantly smaller volume in the occipital lobe, temporal lobe, orbitofrontal gyrus, and rectus gyrus. In the occipital lobe and posterior temporal lobe, these are regions that showed a smaller volume related to cognitive impairment and visuospatial processing deficits (Millington et al., 2017; Chen et al., 2019). Furthermore, the temporal lobe is also vulnerable to vascular abnormalities (De Jong et al., 1999; de Toledo Ferraz Alves et al., 2011). Next, the orbitofrontal gyrus and rectus gyrus are related to emotion and cognitive function (Suzuki et al., 2019; Xu et al., 2019). We also observed a significantly larger volume in the bilateral caudate nucleus, pallidum, and putamen in the severe BG-PVS group. Such volume differences in older adults have not been reported previously. The larger volume of the basal ganglia reflects functional changes in the cortico-basal ganglia-thalamocortical circuit and has been observed in patients with cognitive and behavioral impairment (Alexander and Crutcher, 1990; Frangou et al., 2004; Langen et al., 2007; Sandman et al., 2014).

After covarying CBF with regional GMV, we found a limited spatial overlap between the GMV and CBF pattern. Compared with the mild BG-PVS group, the severe BG-PVS group showed widespread hypoperfusion in the cortex, except for the right amygdala and right anterior middle temporal lobe. Therefore, it was hypothesized that BG-PVS were related to a whole-brain perfusion change rather than localized changes. The association between BG-PVS and CBF is in line with previous studies showing that CBF is negatively related to BG-PVS (Wang et al., 2020). However, the results contradicted with an MRI study of 132 memory clinic patients that observed no association between BG-PVS and total brain perfusion (Onkenhout et al., 2020). This discrepancy could be explained by differences in the study population. The normal cortex has a relatively high CBF to supply the metabolic activity of the cortex (Gregg et al., 2015). The presence of cerebral hypoperfusion can put older subjects at risk for neuronal injuries (Sowell et al., 2003; Gregg et al., 2015). Another study (Toth et al., 2017) found that even mild impairment of CBF can promote cognitive impairment in the elderly. These bodies of evidence led the authors of this study to consider severe BG-PVS, which were previously considered to be present in normal aging, as an abnormality.

The gray matter volume and cerebral blood flow analysis in this study showed that individuals with severe BG-PVS suffered chronic brain atrophy and hypoperfusion. Therefore, it is important to maintain brain health by controlling the severity of BG-PVS. The results revealed that BG-PVS are associated with numerous cardiovascular risk factors (such as age, sex, and hypertensive arteriopathy), consistent with previous studies (Zhu et al., 2010; Kress et al., 2014; Kyrtsos and Baras, 2015; Ramirez et al., 2016). This study also demonstrated the relationship between the severity of BG-PVS and cardiovascular risk burden, suggesting that BG-PVS can be used as an imaging marker to predict cardiovascular risk. Future studies can investigate whether CBF, GMV, and cardiovascular parameters are independently associated with BG-PVS, or whether there are any interaction effects between these different biomarkers.

This study focused on cognitively intact individuals and presented some new findings that might suggest directions for future research. There is a growing body of evidence that cerebral microvascular dysfunction and brain hypoperfusion play critical roles in the pathogenesis of dementia (Kalaria, 2012; Banerjee et al., 2017; Toth et al., 2017). However, the association between baseline BG-PVS and neurodegenerative disease still needs to be verified. In addition, the findings highlight the importance of controlling the cardiovascular risk burden in individuals with severe BG-PVS, which may have both public and clinical significance. This study is crucial for understanding the evolution of BG-PVS-related diseases and can improve future treatment decisions.

There are some limitations to this study. First, the visual scoring system was an observer-dependent task (Dubost et al., 2019). The automated quantification of BG-PVS was more effective and objective than visual scoring (Dubost et al., 2019). Furthermore, the automated quantification had great potential to evaluate the burden of BG-PVS as a continuous rather than categorical measure (Dubost et al., 2019). This would allow for an accurate diagnosis and better monitoring of BG-PVS progression. Second, the investigators used the number of classic BG-PVS to reflect the burden of BG-PVS. However, it was unclear whether single, large, and tumefactive BG-PVS would be more relevant in the clinic than multiple small BG-PVS (Ramirez et al., 2016). Third, FGCRS was based on the European population, which might influence its generalization to the Chinese population. Fourth, cardiovascular risk factors were categorically treated in this study. The relationship between BG-PVS and cardiovascular risk factors can be better elucidated by analyzing cardiovascular disease severity and duration of exposure.

## Conclusion

This study indicated that for cognitively intact older adults, the presence of severe BG-PVS is associated with smaller cortical GMV and CBF, larger subcortical GMV, and a higher cardiovascular risk burden. This study suggested that early identification is crucial for understanding the evolution of BG-PVS-related diseases.

## Data Availability Statement

The datasets generated during the current study are available from the corresponding author on reasonable request.

## Ethics Statement

The studies involving human participants were reviewed and approved by Institutional review board of Peking Union Medical College Hospital. The patients/participants provided their written informed consent to participate in this study.

## Author Contributions

SL, HY, ZZ, and FF: conception and design of the study. SL, BH, YCZ, and CM: acquisition of data. SL, YWZ, and ZZ: analysis and interpretation of data and SL, ZZ, and FF: drafting of the article. All authors contributed to the article and approved the submitted version.

## Conflict of Interest

The authors declare that the research was conducted in the absence of any commercial or financial relationships that could be construed as a potential conflict of interest.

## Publisher's Note

All claims expressed in this article are solely those of the authors and do not necessarily represent those of their affiliated organizations, or those of the publisher, the editors and the reviewers. Any product that may be evaluated in this article, or claim that may be made by its manufacturer, is not guaranteed or endorsed by the publisher.
