# Supplementary material for: The Association Between Perivascular Spaces and Cerebral Blood Flow, Brain Volume, and Cardiovascular Risk
Source: Front Aging Neurosci. 2021 Aug 31;13:599724. doi: 10.3389/fnagi.2021.599724 (PMC8438293; doi:10.3389/fnagi.2021.599724)
Supplement: Supplementary file 1 [file Table_1.docx]

**The association between perivascular spaces, and cerebral blood flow, brain volume and cardiovascular risk**

**Supplementary material 1**. Regional GMV and CBF in the mild BG-PVS group and the severe BG-PVS group.

|  | **GMV (mL)** | | | **CBF (mL/100g/min)** | | |
| --- | --- | --- | --- | --- | --- | --- |
| **Region** | Mild BG-PVS group | Severe BG-PVS group | P_FDR_ | Mild BG-PVS group | Severe BG-PVS group | P_FDR_ |
| Hippocampus L | 2.03±0.23 | 1.99±0.25 | 0.719 | 44.80±6.78 | 43.80±7.27 | 0.016* |
| Hippocampus R | 2.20±0.25 | 2.15±0.26 | 0.621 | 44.54±6.63 | 43.60±7.11 | 0.025* |
| Amygdala L | 1.59±0.19 | 1.57±0.24 | 0.856 | 38.71±5.70 | 37.85±6.15 | 0.018* |
| Amygdala R | 1.75±0.20 | 1.73±0.22 | 0.845 | 38.16±5.80 | 37.53±6.16 | 0.163 |
| AntMedTeLo L | 4.95±0.67 | 4.81±0.72 | 0.131 | 30.53±5.22 | 27.65±4.86 | 0.003* |
| AntMedTeLo R | 4.86±0.62 | 4.66±0.66 | 0.341 | 30.92±5.10 | 28.71±5.36 | 0.065 |
| AntLatTeLo L | 5.84±0.74 | 5.70±0.71 | 0.303 | 30.47±5.51 | 27.23±4.98 | 0.002* |
| AntLatTeLo R | 5.46±0.62 | 5.28±0.60 | 0.138 | 31.93±5.52 | 28.33±5.73 | 0.002* |
| Parahippocampal gyrus L | 3.29±0.37 | 3.23±0.43 | 0.536 | 37.22±5.11 | 35.43±5.52 | 0.011* |
| Parahippocampal gyrus R | 3.32±0.39 | 3.28±0.44 | 0.674 | 37.39±5.18 | 35.11±5.75 | 0.002* |
| Superior temporal gyrus L | 6.16±0.74 | 6.00±0.79 | 0.381 | 48.34±6.37 | 44.39±7.50 | ＜0.001* |
| Superior temporal gyrus R | 6.71±0.77 | 6.51±0.84 | 0.134 | 49.35±6.10 | 45.19±7.16 | ＜0.001* |
| InfMidTemGy L | 8.90±1.08 | 8.86±1.21 | 0.052 | 41.06±6.05 | 37.54±6.12 | ＜0.001* |
| InfMidTemGy R | 9.48±1.06 | 9.15±1.24 | 0.021* | 42.62±5.83 | 38.85±6.45 | ＜0.001* |
| Fusiform gyrus L | 2.81±0.38 | 2.72±0.41 | 0.177 | 33.81±5.25 | 31.48±5.11 | 0.002* |
| Fusiform gyrus R | 2.93±0.39 | 2.83±0.40 | 0.155 | 34.60±5.34 | 32.74±5.41 | 0.010* |
| Insula L | 7.36±0.71 | 7.37±0.83 | 0.773 | 43.02±5.53 | 40.71±5.80 | ＜0.001* |
| Insula R | 7.24±0.70 | 7.32±0.84 | 0.332 | 43.18±5.48 | 40.68±5.76 | ＜0.001* |
| Lateral occipital lobe L | 20.12±2.08 | 19.49±2.35 | 0.021* | 36.27±7.83 | 31.07±7.86 | ＜0.001* |
| Lateral occipital lobe R | 20.28±2.16 | 19.60±2.20 | 0.009* | 36.67±8.10 | 31.11±7.81 | ＜0.001* |
| Anterior cingulate gyrus L | 3.77±0.47 | 3.64±0.54 | 0.097 | 48.02±6.50 | 44.55±6.73 | 0.002* |
| Anterior cingulate gyrus R | 3.85±0.61 | 3.80±0.59 | 0.379 | 48.38±6.82 | 44.67±6.92 | 0.002* |
| Posterior cingulate gyrus L | 4.18±0.47 | 4.10±0.51 | 0.343 | 62.69±8.14 | 58.20±9.15 | ＜0.001* |
| Posterior cingulate gyrus R | 3.95±0.45 | 3.89±0.48 | 0.330 | 64.35±8.62 | 59.09±9.76 | ＜0.001* |
| Middle frontal gyrus L | 18.25±2.11 | 18.48±2.62 | 0.331 | 40.59±6.93 | 36.75±6.23 | ＜0.001* |
| Middle frontal gyrus R | 18.14±1.91 | 18.09±2.22 | 0.943 | 41.76±6.45 | 37.38±5.80 | ＜0.001* |
| PosTeLo L | 21.62±2.12 | 21.10±2.21 | 0.007* | 43.91±6.19 | 39.85±6.63 | ＜0.001* |
| PosTeLo R | 22.26±2.19 | 21.67±2.19 | 0.072 | 43.48±6.30 | 39.65±6,54 | ＜0.001* |
| Inferior lateral parietal lobe L | 16.93±1.76 | 16.60±1.98 | 0.090 | 40.19±6.57 | 36.12±6.83 | ＜0.001* |
| Inferior lateral parietal lobe R | 17.56±1.74 | 17.41±1.97 | 0.324 | 41.33±7.04 | 37.03±6.70 | ＜0.001* |
| Caudate L | 2.93±0.40 | 3.28±0.58 | ＜0.001* | 29.23±7.45 | 28.82±6.55 | 0.242 |
| Caudate R | 2.98±0.39 | 3.41±0.56 | ＜0.001* | 29.94±7.48 | 28.83±6.90 | 0.060 |
| Accumbens L | 0.35±0.04 | 0.34±0.05 | 0.847 | 40.37±8.75 | 40.53±7.60 | 0.488 |
| Accumbens R | 0.31±0.04 | 0.31±0.04 | 0.389 | 39.54±8.80 | 39.13±7.27 | 0.155 |
| Putamen L | 3.70±0.51 | 4.04±0.68 | ＜0.001* | 42.22±7.19 | 44.07±6.75 | 0.975 |
| Putamen R | 3.91±0.47 | 4.25±0.64 | ＜0.001* | 41.46±7.39 | 43.62±6.73 | 0.831 |
| Thalamus L | 5.36±0.47 | 5.16±0.57 | 0.203 | 46.04±9.41 | 42.48±7.99 | 0.012* |
| Thalamus R | 5.29±0.50 | 5.09±0.54 | 0.324 | 44.56±9.99 | 41.92±8.55 | 0.206 |
| Pallidum L | 0.32±0.12 | 0.39±0.15 | 0.001* | 42.81±9.42 | 44.64±8.15 | 0.989 |
| Pallidum R | 0.37±0.12 | 0.43±0.15 | 0.003* | 40.81±8.69 | 43.40±8.00 | 0.390 |
| Precentral gyrus L | 13.28±1.33 | 13.12±1.53 | 0.594 | 40.95±6.02 | 37.83±6.20 | 0.001* |
| Precentral gyrus R | 13.22±1.38 | 13.18±1.65 | 0.893 | 41.81±5.79 | 38.35±5.70 | ＜0.001* |
| Rectal gyrus L | 2.35±0.30 | 2.25±0.31 | 0.043* | 43.04±7.36 | 39.53±6.87 | 0.015* |
| Rectal gyrus R | 2.43±0.31 | 2.34±0.33 | 0.030* | 42.79±7.74 | 39.44±6.56 | 0.012* |
| Orbital frontal gyrus L | 10.18±1.12 | 9.94±1.30 | 0.093 | 34.81±6.63 | 32.16±5.59 | 0.002* |
| Orbital frontal gyrus R | 11.25±1.18 | 10.92±1.30 | 0.008* | 35.55±6.01 | 32.28±5.49 | 0.001* |
| Inferior frontal gyrus L | 8.00±0.84 | 7.85±0.91 | 0.333 | 39.33±6.74 | 36.25±6.80 | 0.020* |
| Inferior frontal gyrus R | 7.89±0.85 | 7.66±0.88 | 0.053 | 42.79±6.29 | 38.59±6.81 | ＜0.001* |
| Superior frontal gyrus L | 26.76±2.71 | 26.71±2.70 | 0.842 | 35.45±5.92 | 32.82±5.26 | 0.001* |
| Superior frontal gyrus R | 25.92±2.53 | 25.89±2.72 | 0.854 | 35.21±6.14 | 32.66±5.63 | 0.003* |
| Postcentral gyrus L | 11.48±1.23 | 11.15±1.36 | 0.051 | 39.86±5.94 | 36.55±6.22 | ＜0.001* |
| Postcentral gyrus R | 11.15±1.23 | 10.88±1.45 | 0.137 | 40.20±5.78 | 36.21±6.09 | ＜0.001* |
| Superior parietal gyrus L | 18.63±1.88 | 18.80±2.46 | 0.449 | 40.37±7.56 | 35.05±7.96 | ＜0.001* |
| Superior parietal gyrus R | 18.64±1.80 | 18.82±2.37 | 0.325 | 41.22±4.84 | 35.17±8.03 | ＜0.001* |
| Lingual gyrus L | 6.70±0.74 | 6.49±0.95 | 0.408 | 45.40±8.19 | 39.53±9.34 | ＜0.001* |
| Lingual gyrus R | 6.62±0.77 | 6.35±0.82 | 0.064 | 47.23±8.47 | 41.58±9.92 | ＜0.001* |
| Cuneus L | 4.76±0.66 | 4.56±0.69 | 0.169 | 40.40±9.39 | 34.10±10.31 | ＜0.001* |
| Cuneus R | 4.55±0.65 | 4.31±0.68 | 0.090 | 42.20±9.73 | 35.70±10.88 | ＜0.001* |

Note: Other than P_FDR_ values, data are means ± standard deviation

AntMedTeLo = Anterior middle temporal lobe, AntLatTeLo = Anterior lateral temporal lobe, InfMidTemGy = Inferior middle temporal gyrus, PosTeLo = Posterior temporal lobe. GMV, grey matter volume; CBF, cerebral blood flow; BG-PVS, basal ganglia perivascular spaces; L, left; R, right; P_FDR_: False discovery rate-corrected p-values

*Significant at p _FDR_ ＜ 0.05; this difference was significant
